# Neutrophil-to-Lymphocyte Ratio in Rectal Cancer—Novel Biomarker of Tumor Immunogenicity During Radiotherapy or Confounding Variable?

**DOI:** 10.3390/ijms20102448

**Published:** 2019-05-17

**Authors:** Lore Helene Braun, David Baumann, Kerstin Zwirner, Ewald Eipper, Franziska Hauth, Andreas Peter, Daniel Zips, Cihan Gani

**Affiliations:** 1 Department of Radiation Oncology, University Hospital and Medical Faculty Tübingen, Eberhard Karls University Tübingen, 72076 Tübingen, Germany; helene.braun@med.uni-tuebingen.de (L.H.B.); David.baumann@med.uni-tuebingen.de (D.B.); Kerstin.zwirner@med.uni-tuebingen.de (K.Z.); Franziska.hauth@med.uni-tuebingen.de (F.H.); Daniel.zips@med.uni-tuebingen.de (D.Z.); 2 Institute for Clinical Chemistry and Pathobiochemistry, University Hospital Tübingen, 72076 Tübingen, Germany; ewald.eipper@med.uni-tuebingen.de (E.E.); Andreas.peter@med.uni-tuebingen.de (A.P.); 3 German Cancer Research Center (DKFZ), Heidelberg and German Cancer Consortium (DKTK), 69120 Heidelberg, Germany; 4 Gastrointestinal Cancer Center, Comprehensive Cancer Center Tübingen-Stuttgart, 72076 Tübingen, Germany

**Keywords:** rectal cancer, inflammation, leukocytosis, neutrophil-to-lymphocyte ratio, pathologic response, neoadjuvant radiotherapy, tumor volume

## Abstract

The aim of this study was to investigate the predictive value of blood-derived makers of local and systemic inflammatory responses on early and long-term oncological outcomes. A retrospective analysis of patients with locally advanced rectal cancer treated with preoperative long-course 5-fluorouracil-based radiochemotherapy was performed. Differential blood counts before neoadjuvant treatment were extracted from the patients’ electronic charts. Optimal cut-off values for neutrophil-to-lymphocyte ratio (NLR), platelet-to-lymphocyte ratio (PLR), and lymphocyte-to-monocyte ratio (LMR) were determined. Potential clinical and hematological prognostic factors for disease-free survival (DFS) were studied using uni- and multivariate analysis. A total of 220 patients were included in the analysis. Median follow-up was 67 months. Five-year DFS and overall survival (OS) were 70% and 85%, respectively. NLR with a cut-off value of 4.06 was identified as optimal to predict DFS events. In multivariate analysis, only tumor volume (HR 0.33, 95% CI (0.14–0.83), *p* = 0.017) and NLR (HR 0.3, 95% CI (0.11–0.81), *p* = 0.017) remained significant predictors of DFS. Patients with a good histological response (Dworak 3 and 4) to radiotherapy also had a lower NLR than patients with less pronounced tumor regression (3.0 vs. 4.2, *p* = 0.015). A strong correlation between primary tumor volume and NLR was seen (Pearson’s *r* = 0.64, *p* < 0.001). Moreover, patients with T4 tumors had a significantly higher NLR than patients with T1–T3 tumors (6.6 vs. 3.3, *p* < 0.001). An elevated pretherapeutic NLR was associated with higher T stage, inferior DFS, and poor pathological response to neoadjuvant radiochemotherapy. A strong correlation between NLR and primary tumor volume was seen. This association is important for the interpretation of study results and for the design of translational studies which are warranted.

## 1. Introduction

Today, the standard of care for locally advanced rectal cancer (LARC) consists of neoadjuvant radio(chemo)therapy followed by radical resection. The evolution from surgery as the only treatment to a multimodal concept has resulted in excellent oncological outcomes [1,2]. There are some known unfavorable prognostic factors indicating a higher risk of local and systemic relapse, such as high tumor stage, lymph node metastasis, R1 or R2 resection, lymphovascular invasion, and poor pathological response to neoadjuvant treatment [2,3]. Still, patients with comparable risk profiles can have a wide variation of oncological outcomes. An important factor contributing to this phenomenon might be the differential interaction between the individual tumor microenvironment and the patient’s immune system [4,5]. In recent years, evidence has grown that local and systemic inflammatory responses are associated with inferior outcomes for solid tumors like renal cell carcinoma, hepatocellular carcinoma, or gastric and esophageal cancer [6,7]. This tumor-related inflammatory response can be reflected by hematological parameters like, amongst others, a high leukocyte count, elevated C-reactive protein (CRP), or the ratios of different cell types, e.g., the neutrophil-to-lymphocyte ratio (NLR) [8]. This was confirmed by several, mainly retrospective studies [9,10,11,12,13,14,15]. However, to our knowledge, tumor burden has not been differentially evaluated in uni- and multivariate analyses.

Therefore, the aim of this study was to investigate the prognostic value of pretherapeutic blood-derived hematological parameters on early and long-term oncological results in patients with LARC, considering the primary tumor volume as a potentially confounding variable.

## 2. Results

A total of 220 patients with UICC (Union Internationale Contre le Cancer, 8th edition) stage II or III rectal cancer (64.5% male, 35.5% female) with a median age of 65.5 years were treated during the defined timeframe. Median follow-up was 67 months. Patient- and treatment-related parameters are summarized in Table 1 and Table 2. Baseline hematological parameters are shown in Table 3; for 108 patients, a baseline differential blood count was available.

For the entire group, five-year local control, distant control, disease-free survival (DFS), and overall survival (OS) were 92%, 79%, 70%, and 85%, respectively. For DFS of the entire group, the area under the curve on receiver operator characteristics (ROC) analysis was 0.60, 0.57, and 0.42 for the ratios of neutrophil-to-lymphocyte, platelet-to-lymphocyte, and lymphocyte-to-monocyte, respectively. We therefore included the NLR in subsequent analyses. Youden’s test revealed an NLR of 4.06 as optimal to predict DFS events. On univariate analysis of pretherapeutically available parameters, DFS was significantly worse in patients with larger primary tumors (*p* = 0.003), an elevated NLR (*p* = 0.001), an elevated neutrophil count (*p* = 0.019), and an elevated leucocyte count (*p* = 0.023). All variables with *p* < 0.1 in univariate analysis (T-stage, gross tumor volume (GTV), NLR, absolute neutrophil and leukocyte counts) were included in the multivariate analysis for DFS. Among these, only tumor volume (HR 0.33, 95% CI (0.14–0.83), *p* = 0.017) and NLR (HR 0.3, 95% CI (0.11–0.81), *p* = 0.017) remained significant on multivariate analysis (Table 4).

**Figure 1:**
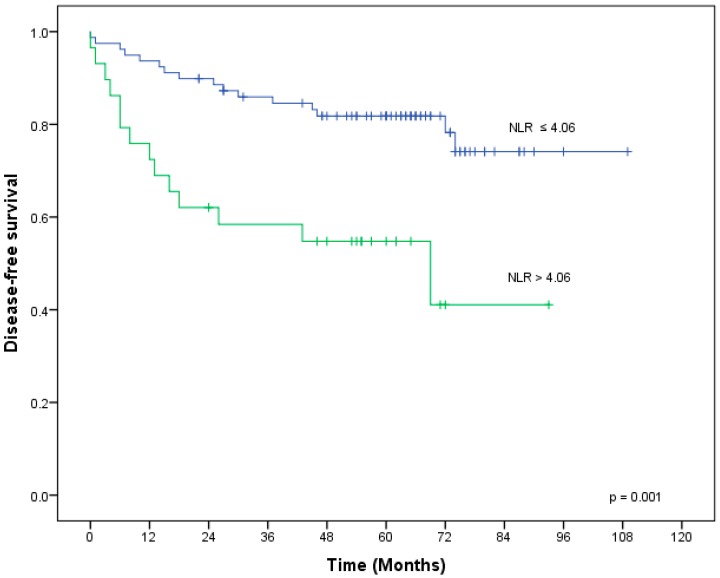
Disease-free survival in relation to NLR.

Figure 1 shows DFS in relation to NLR. Patients with a histologically good response (Dworak regression grade 3–4) to radiotherapy also had a lower NLR than patients with less pronounced tumor regression (Dworak regression grade 0–2) (3.0 vs. 4.2, *p* = 0.015).

**Figure 2:**
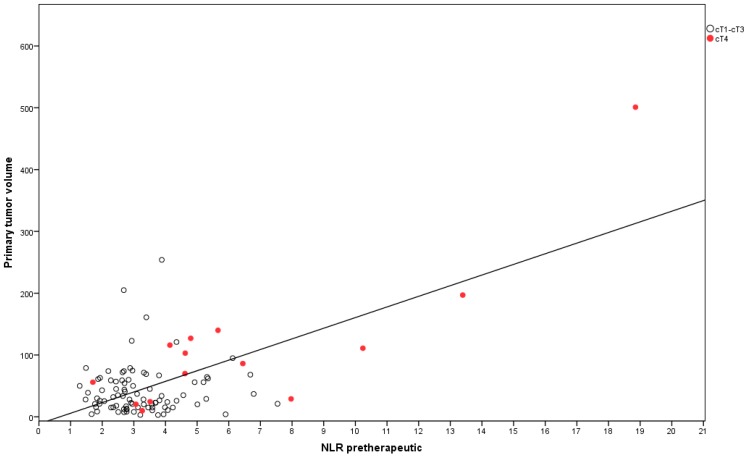
Dot plot showing a correlation between primary tumor volume and NLR.

A strong correlation between primary tumor volume and NLR was seen (Pearson’s *r* = 0.64, *p* < 0.001). Moreover, patients with T4 tumors had a significantly higher NLR than patients with T1–T3 tumors (6.6 vs. 3.3, *p* < 0.001), as depicted in Figure 2.

## 3. Discussion

Cancer-related inflammation is a field of growing scientific interest. In an update of “The Hallmarks of Cancer”, “avoiding immune destruction” and “tumor-promoting inflammation” have been accepted as an emerging hallmark and an enabling characteristic of cancer, respectively [16,17].

In the present study, we investigated the impact of immune cells as a marker of an inflammatory state on oncological results after radiochemotherapy in LARC. To the best of our knowledge, our study is the first to include the actual tumor volume in the statistical analyses.

The local interaction of tumor cells with immune cells such as neutrophils, myeloid-derived suppressor cells, macrophages, and lymphocytes, together with endothelial and stromal cells, can have both pro- and antitumoral effects. In this context, a higher degree of infiltrating lymphocytes has been established as a positive prognostic factor, whereas a high intratumoral neutrophil count has been found to be an independent negative prognostic factor for pathologic response, DFS, and OS in colorectal carcinoma [12,18,19,20,21].

There is a complex interaction between local cancer-related immune response and systemic immune reaction, as both immune and tumor cells can secrete cytokines and chemokines into the systemic circulation. This communication of the tumor with other organs such as the bone marrow or the spleen can lead to aberrant myelopoiesis, resulting in further recruitment of immune cells into the tumor microenvironment [19,20]. Hereby, a high systemic count of myeloid-derived immune cells such as monocytes, macrophages, and neutrophils has been associated with an inferior prognosis and tumor progression, in contrast to a high peripheral number of lymphocytes [19,20,22]. Especially, the ratio of myeloid-derived immune cells as potential tumor-favoring counterparts of lymphocytes with potential antitumor action can serve as a predictive tool in patients with otherwise equally distributed established risk factors. In a systematic review including over 10,000 patients with advanced rectal cancer, an elevated pretherapeutic NLR has been found to correlate with poor cancer-specific and overall survival [9]. Accordingly, in our study cohort, patients with an elevated pretherapeutic NLR (>4.06) showed an inferior DFS in multivariate analysis. This cut-off is in line with previously reported cut-off values for NLR [9,13,23,24]. In addition to NLR, only pretherapeutic tumor volume was significantly associated with DFS in multivariate analysis. Furthermore, a higher pretherapeutic NLR was associated with a lower probability of achieving a good pathological response to neoadjuvant therapy, confirming the relation between tumor-associated inflammation and radioresistance, as previously described [11,12,13,14,25,26]. One could envision personalizing treatments according to the baseline NLR, for instance by intensifying a treatment via dose-escalated radiotherapy or intensified systemic treatment in patients with an elevated NLR. Alternatively, patients with a very favorable NLR might have a higher chance to achieve a clinical complete response and could be managed non-operatively, a concept which is currently tested in several clinical trials [27].

Interestingly, we found a strong association between NLR and primary tumor volume, with T4 tumors having the highest NLR. This finding is in accordance with the results of Kim et al., who found an association of both a higher NLR and a larger tumor diameter in pretherapeutic radiologic staging with a lower probability of achieving a good histological response. In their study, a poor histological response and a higher pretherapeutic NLR were significantly associated with an inferior cancer-specific survival [26]. This finding could indicate that the NLR might be influenced by the primary tumor volume, which itself is a negative prognostic factor [2,3,28].

There are a couple of studies that found a correlation of both higher clinical T stage and NLR with either pathologic response to neoadjuvant therapy [26] or long-term oncological outcome [9,12,15]. In a systematic review by Haram et al., a higher NLR was associated with a higher T stage or stage III–IV disease. This finding could be confirmed by our cohort, which showed a significantly higher NLR in patients with T4 tumors compared to patients with T1–T3 tumors (Figure 2). A higher degree of local infiltration of the tumor and surrounding tissue by neutrophils and other myeloid cells and consecutive remodeling of their activity by the tumor microenvironment can lead to enhanced proliferation, angiogenesis, and metastatic potential of the tumor. This might result in higher T-stage and advanced-stage disease as well as worse pathological response and eventually inferior prognosis [19,21,29]. The inflammatory response in the tumor microenvironment maintains the systemic inflammatory reaction, leading to sustained high levels of inflammatory markers and immune cells. Liu et al. found an association between absolute number of systemic lymphocytes and pathologic response; furthermore, they showed that a higher peripheral lymphocyte count was associated with a higher infiltration of CD4+ and CD8+ cells into the tumor [18]. Likewise, an effective local or systemic treatment of tumors leads to a decline of systemic inflammatory markers, so that a link between tumor mass and the degree of systemic inflammatory response, which our data emphasize, seems reasonable [19,29,30,31,32]. The knowledge of this association is important for the interpretation of the study results and the design of translational studies that investigate the underlying mechanisms.

## 4. Materials and Methods

In this retrospective study, we included patients with locally advanced rectal cancer treated with preoperative long-course 5-fluorouracil-based radiochemotherapy between 2006 and 2013. Treatment-related details were described in a preceding study with an overlapping patient cohort [33]. Patient and tumor characteristics, follow-up data, and differential blood counts before and during treatment were extracted from the patients’ electronic charts using “Swisslab Laboratory information technology systems” (Nexus AG, Donaueschingen, Germany). Blood cell count and white blood cell differentiation were performed using the flow cytometry-based ADVIA 2120 Hematology system. Serum concentrations of carcinoembryonic antigen were determined using the ADVIA Centaur immunoassay system, and plasma concentrations of C-reactive protein were measured on the ADVIA 1800 clinical chemistry analyzer using the wide-range assay (all instruments from Siemens Healthineers, Eschborn, Germany). The study was approved by the institutional review board of the Medical Faculty in Tübingen/Germany (Ethik-Kommission an der Medizinischen Fakultät der Eberhard-Karls-Universität und am Uniklinikum Tübingen, 21 December 2015, Identification Code: 733/2015BO1).

Kaplan–Meier survival estimates were calculated from the last fraction of radiotherapy. Death of any cause and local and distant treatment failures were considered events for DFS estimation. Local control and distant control were calculated on a “first-event” basis. Median follow-up was calculated using the reverse Kaplan–Meier method [34]. The log-rank test was used to compare groups in terms of survival. Multivariate analysis was carried out using Cox-regression. ROC were created in order to determine the accuracy of blood cell components and the neutrophil-to-lymphocyte, platelet-to-lymphocyte, and lymphocyte-to-monocyte ratios to predict treatment failures and to define optimal cut-offs using the Youden test. In order to define the volume of the primary tumor, the “gross tumor volume” structure in the planning computed tomography was reviewed by C.G. or L.H.B and re-contoured if required. Oncentra masterplan was used for tumor delineation. Tumor regression was classified according to Dworak [35,36].

The distribution of categorical variables was studied by the Chi-square test. Correlations between linear variables were studied according to Pearson, with a correlation coefficient >0.5 indicating a strong correlation. Student’s t-Test and Mann–Whitney’s U-Test were used to compare groups on the basis of the presence of a normal distribution. A *p*-value <0.05 was considered significant for all tests. All statistical analyses were performed in SPSS 25 (IBM, Armonk, New York, NY, USA).

## 5. Conclusions

In this study cohort, patients with an elevated pretherapeutic NLR (>4.06) showed a poor pathological response to neoadjuvant 5-FU-based radiochemotherapy as well as an inferior DFS in multivariate analysis. Furthermore, there was a strong correlation between NLR and tumor burden. A higher tumor burden was significantly associated with poor DFS in multivariate analysis. T4 tumors had a higher NLR compared to T1–T3 tumors.

There appears to be a strong link between tumor-associated immune response, the effectiveness of local and systemic therapies, and eventually the oncological outcome of the patients. Preclinical and clinical data demonstrate this link and provide a rational on how immune cells might promote aggressiveness, invasiveness, and metastatic potential of tumor cells. Yet, the strong correlation between NLR and tumor volume raises the question whether parameters like the NLR are causative of the inferior prognosis or only a result of larger tumors triggering an inflammatory response in patients with advanced tumors. Therefore, we strongly emphasize the need for well-designed translational studies evaluating systemic inflammation cells and markers and intratumoral immune reaction in pre- and post-therapeutic histological specimens and oncological outcomes.

## Figures and Tables

**Table 1:** Patient- and tumor-related parameters.

		Median	IQR
Age (years)		65.5	15
Tumor location (cm)		6	6
CEA (mg/dL)		3.88	8,7
Primary tumor volume (cc)		33.86	48
		***n***	**%**
Gender	Male	142	64.5
	Female	78	35.5
T-stage	T1	1	0.5
	T2	7	3.2
	T3	193	87.7
	T4	19	8.6
N-stage	cN0	39	17.7
	cN+	181	82.3
Grading	G1	17	7.7
	G2	173	78.6
	G3	24	10.9
	Missing	6	2.7
Location	Lower third	92	41.8
	Mid third	115	52.3
	Upper third	13	5.9

IQR: Interquartile range, CEA: carcinoembryonic antigen.

**Table 2:** Treatment-related parameters.

	Subgroups	*n*	%
Chemotherapy dose	Complete	208	94.5
	Incomplete	12	5.5
Type of surgery	LAR	158	71.8
	APR	62	28.2
TME Quality	Perfect	66	30.0
	Intermediate	17	7.7
	Poor	7	3.2
	Missing	130	59.1
Postoperative T-stage	ypT0	36	16.4
	ypT1	16	7.3
	ypT2	66	30.0
	ypT3	94	42.7
	ypT4	8	3.6
Postoperative N-stage	ypN0	164	74.5
	ypN1	38	17.3
	ypN2	18	8.2
Resection status	R0	214	97.3
	R1	5	2.3
	Rx	1	0.5
Dworak regression	0	27	12.3
	1	70	31.8
	2	1	0.5
	3	44	20.0
	4	35	15.9
	missing	43	19.5
Postoperative Chemotherapy	yes	122	55.5
	no	40	18.2
	missing	58	26.4

TME: Total mesorectal excision.

**Table 3:** Baseline blood counts.

	*n*	Median	Min	Max	IQR
CRP	191	0.26	0.01	11.79	0.63
White cell count	217	7840	4080	18,920	2570
Platelets	216	297	147	810	123.25
Neutrophils	109	5368	808	15,673	2062.69
Lymphocytes	108	1536	521	4438	778.49
Monocytes	108	486	185	1091	186.32
NLR	108	3.11	1.23	18.85	1.71
LMR	108	3.32	1.14	8.08	1.97
NMR	108	11.04	6.35	28.67	4.45
PLR	108	0.18	0.05	0.98	0.13

CRP: C-reactive protein, NLR: neutrophil-to-lymphocyte ratio, LMR: lymphocyte-to-monocyte ratio, NMR: neutrophil-to-monocyte ratio, PLR: platelet-to-lymphocyte ratio.

**Table 4:** Univariate and multivariate analysis for disease-free survival (DFS).

Univariate Analysis			Multivariate Analysis		
Primary tumor location	Lower third vs. upper/mid third	*p*	HR	95% CI	*p*
		0.725	-	-	-
T-stage	cT1/cT2/cT3 vs. cT4	0.087	1.19	0.43–3.30	0.733
N-stage	cN0 vs. cN+	0.571	-	-	-
**GTV**	**≤33.86 cc vs. >33.86 cc**	**0.003**	**0.338**	**0.14–0.83**	**0.017**
Age	≤ 65.5 vs. > 65.5	0.139	-	-	-
CEA	≤ 3.88 vs. > 3.88	0.16	-	-	-
Grading	G3 vs. G1/G2	0.133	-	-	-
CRP	≤ 0.26 vs. > 0.26	0.324	-	-	-
**NLR**	**≤ 4.06 vs. > 4.06**	**0.001**	**0.3**	**0.11–0.81**	**0.017**
Neutrophil count	≤ 6021 vs. > 6021	0.019	2.195	0.61–7.86	0.227
Leukocyte count	≤ 8120 vs. > 8120	0.023	0.375	0.13–1.10	0.074

GTV: gross tumor volume; The bold was added to highlight the only two statistically significant variables in multivariate analysis.

## References

[1] Mendenhall W.M., Bland K.I., Copeland E.M., Summers G.E., Pfaff W.W., Souba W.W., Million R.R. (1992). Does preoperative radiation therapy enhance the probability of local control and survival in high-risk distal rectal cancer?. Ann. Surg..

[2] Rödel C., Graeven U., Fietkau R., Hohenberger W., Hothorn T., Arnold D., Hofheinz R.-D., Ghadimi M., Wolff H.A., Lang-Welzenbach M. (2015). Oxaliplatin added to fluorouracil-based preoperative chemoradiotherapy and postoperative chemotherapy of locally advanced rectal cancer (the German CAO/ARO/AIO-04 study): final results of the multicentre, open-label, randomised, phase 3 trial. Lancet. Oncol..

[3] Maas M., Nelemans P.J., Valentini V., Das P., Rödel C., Kuo L.-J., Calvo F.A., García-Aguilar J., Glynne-Jones R., Haustermans K. (2010). Long-term outcome in patients with a pathological complete response after chemoradiation for rectal cancer: a pooled analysis of individual patient data. Lancet Oncol..

[4] Fridlender Z.G., Sun J., Kim S., Kapoor V., Cheng G., Ling L., Worthen G.S., Albelda S.M. (2009). Polarization of tumor-associated neutrophil phenotype by TGF-beta: N1 versus N2 TAN. Cancer Cell.

[5] Souto J.C., Vila L., Brú A. (2009). Polymorphonuclear neutrophils and cancer: Intense and sustained neutrophilia as a treatment against solid tumors. Med. Res. Rev..

[6] Shen J., Zhu Y., Wu W., Zhang L., Ju H., Fan Y., Zhu Y., Luo J., Liu P., Zhou N. (2017). Prognostic Role of Neutrophil-to-Lymphocyte Ratio in Locally Advanced Rectal Cancer Treated with Neoadjuvant Chemoradiotherapy. Med. Sci. Monit..

[7] Donskov F. (2013). Immunomonitoring and prognostic relevance of neutrophils in clinical trials. Semin. Cancer Biol..

[8] Dumitru C.A., Moses K., Trellakis S., Lang S., Brandau S. (2012). Neutrophils and granulocytic myeloid-derived suppressor cells: immunophenotyping, cell biology and clinical relevance in human oncology. Cancer Immunol. Immunother..

[9] Haram A., Boland M.R., Kelly M.E., Bolger J.C., Waldron R.M., Kerin M.J. (2017). The prognostic value of neutrophil-to-lymphocyte ratio in colorectal cancer: A systematic review. J. Surg. Oncol..

[10] Lee Y.J., Lee S.B., Beak S.K., Han Y.D., Cho M.S., Hur H., Lee K.Y., Kim N.K., Min B.S. (2018). Temporal changes in immune cell composition and cytokines in response to chemoradiation in rectal cancer. Sci. Rep..

[11] Kim T.G., Park W., Kim H., Choi D.H., Park H.C., Kim S.-H., Cho Y.B., Yun S.H., Kim H.C., Lee W.Y. (2018). Baseline neutrophil–lymphocyte ratio and platelet–lymphocyte ratio in rectal cancer patients following neoadjuvant chemoradiotherapy. Tumori J..

[12] Zhang X., Li J., Peng Q., Huang Y., Tang L., Zhuang Q., Lin F., Lin X., Du K., Wu J. (2019). Association of markers of systemic and local inflammation with prognosis of patients with rectal cancer who received neoadjuvant radiotherapy. Cancer Manag. Res..

[13] Ward W.H., Goel N., Ruth K.J., Esposito A.C., Lambreton F., Sigurdson E.R., Meyer J.E., Farma J.M. (2018). Predictive value of leukocyte- and platelet-derived ratios in rectal adenocarcinoma. J. Surg. Res..

[14] Vallard A., Garcia M.-A., Diao P., Espenel S., De Laroche G., Guy J.-B., Mrad M.B., Rancoule C., Kaczmarek D., Muron T. (2018). Outcomes prediction in pre-operative radiotherapy locally advanced rectal cancer: leucocyte assessment as immune biomarker. Radiat. Oncol..

[15] Shen L., Zhang H., Liang L., Li G., Fan M., Wu Y., Zhu J., Zhang Z. (2014). Baseline neutrophil-lymphocyte ratio (≥2.8) as a prognostic factor for patients with locally advanced rectal cancer undergoing neoadjuvant chemoradiation. Radiat. Oncol..

[16] Hanahan D., Weinberg R. (2011). A Hallmarks of Cancer: The Next Generation. Cell.

[17] Hanahan D., Weinberg R.A. (2000). The Hallmarks of Cancer. Cell.

[18] Liu H., Wang H., Wu J., Wang Y., Zhao L., Li G., Zhou M. (2019). Lymphocyte nadir predicts tumor response and survival in locally advanced rectal cancer after neoadjuvant chemoradiotherapy: Immunologic relevance. Radiother. Oncol..

[19] Diakos C.I., Charles K.A., McMillan D.C., Clarke S.J. (2014). Cancer-related inflammation and treatment effectiveness. Lancet Oncol..

[20] Nakamura K., Smyth M.J. (2017). Targeting cancer-related inflammation in the era of immunotherapy. Immunol. Cell Biol..

[21] Rao H.-L., Chen J.-W., Li M., Xiao Y.-B., Fu J., Zeng Y.-X., Cai M.-Y., Xie D. (2012). Increased intratumoral neutrophil in colorectal carcinomas correlates closely with malignant phenotype and predicts patients’ adverse prognosis. PLoS ONE.

[22] Balkwill F.R., Mantovani A. (2012). Cancer-related inflammation: Common themes and therapeutic opportunities. Semin. Cancer Biol..

[23] Carruthers R., Tho L.M., Brown J., Kakumanu S., Mccartney E., Mcdonald A.C. (2012). Systemic inflammatory response is a predictor of outcome in patients undergoing preoperative chemoradiation for locally advanced rectal cancer. Color. Dis..

[24] Krauthamer M., Rouvinov K., Ariad S., Man S., Walfish S., Pinsk I., Sztarker I., Charkovsky T., Lavrenkov K. (2013). A study of inflammation-based predictors of tumor response to neoadjuvant chemoradiotherapy for locally advanced rectal cancer. Oncology.

[25] Ren D.-L., Li J., Yu H.-C., Peng S.-Y., Lin W.-D., Wang X.-L., Ghoorun A., Luo Y.-X. (2019). Nomograms for predicting pathological response to neoadjuvant treatments in patients with rectal cancer. World J. Gastroenterol..

[26] Kim I.Y., You S.H., Kim Y.W. (2014). Neutrophil-lymphocyte ratio predicts pathologic tumor response and survival after preoperative chemoradiation for rectal cancer. BMC Surg..

[27] Gani C., Bonomo P., Zwirner K., Schroeder C., Menegakis A., Rödel C., Zips D. (2017). Organ preservation in rectal cancer—Challenges and future strategies. Clin. Transl. Radiat. Oncol..

[28] Wen B., Zhang L., Wang C., Huang R., Peng H., Zhang T., Dong J., Xiao W., Zeng Z., Liu M. (2015). Prognostic significance of clinical and pathological stages on locally advanced rectal carcinoma after neoadjuvant chemoradiotherapy. Radiat. Oncol..

[29] Gabrilovich D.I., Ostrand-Rosenberg S., Bronte V. (2012). Coordinated regulation of myeloid cells by tumours. Nat. Rev. Immunol..

[30] Diefenhardt M., Hofheinz R.-D., Martin D., Beißbarth T., Arnold D., Hartmann A., von der Grün J., Grützmann R., Liersch T., Ströbel P. (2019). Leukocytosis and neutrophilia as independent prognostic immunological biomarkers for clinical outcome in the CAO/ARO/AIO-04 randomized phase 3 rectal cancer trial. Int. J. Cancer.

[31] Diaz-Montero C.M., Salem M.L., Nishimura M.I., Garrett-Mayer E., Cole D.J., Montero A.J. (2009). Increased circulating myeloid-derived suppressor cells correlate with clinical cancer stage, metastatic tumor burden, and doxorubicin-cyclophosphamide chemotherapy. Cancer Immunol. Immunother..

[32] Ohki S., Shibata M., Gonda K., Machida T., Shimura T., Nakamura I., Ohtake T., Koyama Y., Suzuki S., Ohto H. (2012). Circulating myeloid-derived suppressor cells are increased and correlate to immune suppression, inflammation and hypoproteinemia in patients with cancer. Oncol. Rep..

[33] Gani C., Schroeder C., Heinrich V., Spillner P., Lamprecht U., Berger B., Zips D. (2016). Long-term local control and survival after preoperative radiochemotherapy in combination with deep regional hyperthermia in locally advanced rectal cancer. Int. J. Hyperth..

[34] Schemper M., Smith T.L. (1996). A note on quantifying follow-up in studies of failure time. Control. Clin. Trials.

[35] Thies S., Langer R. (2013). Tumor Regression Grading of Gastrointestinal Carcinomas after Neoadjuvant Treatment. Front. Oncol..

[36] Dworak O., Keilholz L., Hoffmann A. (1997). Pathological features of rectal cancer after preoperative RCT. Int. J. Colorectal Dis..

